# Delayed serum sickness-like reaction mimicking septic shock after antivenom therapy: A case report

**DOI:** 10.1097/MD.0000000000051039

**Published:** 2026-10-23

**Authors:** Xiaobin Wang, Wenkai Bin

**Affiliations:** a Department of Emergency Medicine, Luzhou Hospital of Traditional Chinese Medicine, Luzhou, Sichuan, China; b Animal Injury and Poisoning Treatment Center, Nanhua Hospital Affiliated to University of South China, Hengyang, Hunan, China.

**Keywords:** antivenom, case report, procalcitonin, septic shock, serum sickness-like reaction, snakebite

## Abstract

**Rationale:**

Antivenom is the key specific treatment for clinically significant snakebite envenoming. Delayed serum sickness-like reactions after antivenom therapy may be overlooked after discharge and can resemble infection or septic shock.

**Patient concerns:**

A woman in her early 60s returned approximately 7 days after antivenom therapy with generalized urticarial and purpuric maculopapular rash, pruritus, fever, vomiting, fatigue, dizziness, hypotension, and thrombocytopenia.

**Diagnoses:**

The patient was initially managed for suspected sepsis and septic shock because of circulatory instability, elevated high-sensitivity C-reactive protein, elevated serum amyloid A, elevated procalcitonin, and inflammatory changes on chest computed tomography. The timing after antivenom exposure, rash pattern, decreased complement C3 and C4, proteinuria, and subsequent clinical course supported a severe delayed serum sickness-like reaction as an important contributor. Infection could not be completely excluded because systematic pathogen testing was not completed during the acute phase.

**Interventions:**

The patient received empirical anti-infective therapy, vasopressor support, fluid resuscitation, hydrocortisone, ebastine, albumin supplementation, and other supportive care.

**Outcomes:**

After 7 days of treatment, blood pressure stabilized, rash and pruritus largely resolved, platelet count and inflammatory markers improved, and follow-up laboratory findings showed substantial recovery.

**Lessons:**

Delayed antivenom-related serum sickness-like reactions may mimic septic shock and may be accompanied by elevated procalcitonin. In patients who develop rash and hypotension within days after antivenom therapy, clinicians should treat possible sepsis promptly while reassessing antivenom exposure, skin findings, complement levels, urinalysis, microbiological evidence, and treatment response.

## 1. Introduction

Snakebite envenoming remains an important emergency condition in many regions, and antivenom is the main specific therapy for clinically significant envenoming.^[1]^ Immediate hypersensitivity reactions to antivenom are widely recognized during or shortly after infusion, but delayed reactions are more likely to appear after discharge and may therefore be missed or attributed to other causes.^[2]^ Delayed reactions after antivenom administration can include rash, fever, pruritus, arthralgia, proteinuria, and complement consumption; severe cases may present with systemic inflammatory manifestations and circulatory instability.^[3–5]^

The diagnostic problem becomes clinically important when delayed immune-mediated manifestations overlap with findings that suggest infection. Fever, hypotension, thrombocytopenia, pulmonary inflammatory changes, and elevated procalcitonin (PCT) can reasonably lead clinicians to treat suspected sepsis or septic shock.^[6,7]^ However, recent antivenom exposure, urticarial or purpuric skin lesions, decreased complement levels, proteinuria, and improvement after anti-allergic and glucocorticoid treatment may suggest a serum sickness-like reaction. We report this case to highlight a practical bedside challenge: a delayed antivenom-related immune reaction that mimicked septic shock and was confounded by markedly elevated PCT. This case report was prepared in accordance with the CARE guideline.

## 2. Case presentation

### 2.1. Patient information and initial presentation

A woman in her early 60s was admitted on August 24, 2025, approximately 1 hour after a snakebite, because of pain and swelling of the left hand. The snake was not captured, and species identification was therefore not possible. Based on the patient’s description, local snakebite epidemiology, progression of local swelling, and laboratory findings, the envenoming was clinically considered compatible with a pit viper bite, possibly *Protobothrops mucrosquamatus*. Because no species-specific antivenom is currently available in China for *P mucrosquamatus*, *Deinagkistrodon acutus* antivenom was administered according to guideline recommendations for suspected *P mucrosquamatus* envenoming.

### 2.2. Clinical findings at first admission

At the first admission, physical examination showed a body temperature of 36.5°C, a pulse rate of 52 beats/min, a respiratory rate of 21 breaths/min, and a blood pressure of 128/61 mm Hg. Marked swelling and tenderness involved the distal to proximal two-thirds of the left upper limb. A puncture-like wound was present in the first web space of the left hand, with hemorrhagic blisters around it. Initial laboratory tests showed a neutrophil percentage of 80.7% and a high-sensitivity C-reactive protein (hs-CRP) level of 3.64 mg/L. Coagulation function, liver and kidney function, electrolytes, and infection-related markers showed no obvious abnormalities.

### 2.3. Initial therapeutic intervention

Within 1 hour after admission, 2 vials of *D acutus* antivenom were administered intravenously. The skin test was negative, no premedication was given, and no immediate reaction occurred during or after infusion. The patient also underwent local incision for decompression and drainage and received anti-inflammatory treatment, mannitol for swelling reduction, fluid replacement, tetanus prophylaxis, and symptomatic supportive care. After local swelling and pain improved, she was discharged on August 28, 2025.

### 2.4. Delayed symptoms and emergency reassessment

On August 31, 2025, approximately 7 days after antivenom administration, the patient developed a generalized urticarial rash, pruritus, vomiting, poor appetite, and fatigue. She returned to the emergency department on September 1. During observation, the rash and pruritus worsened, and her maximum temperature reached 39.3°C. That night, she developed dizziness, hypotension, and bradycardia. Despite fluid resuscitation and vasopressor treatment, her systolic blood pressure fluctuated between 70 and 90 mm Hg. She was admitted on September 2 with a preliminary diagnosis of serum sickness and shock of undetermined cause.

### 2.5. Clinical findings at second admission

At the second admission, the patient was conscious but appeared unwell. Her body temperature was 36.5°C, pulse rate was 86 beats/min, respiratory rate was 26 breaths/min, and blood pressure was 84/55 mm Hg while she was receiving norepinephrine at 0.05 μg/kg/min. Her oxygen saturation was 98%. Diffuse red to violaceous maculopapular eruptions were present over the body, some of which had coalesced into non-blanching patches, predominantly on both lower limbs. Urticarial lesions were observed on the chest and abdomen, and mild edema was present in both lower limbs. No definite focal positive findings were detected on cardiac, pulmonary, or abdominal examination.

### 2.6. Diagnostic assessment

Laboratory tests during the second hospitalization showed a white blood cell count of 12.56 × 10^9^/L, a neutrophil percentage of 88.8%, hemoglobin of 110 g/L, and a platelet count of 62 × 10^9^/L. The hs-CRP level was 227.94 mg/L, serum amyloid A was 285.85 mg/L, and PCT was 6.230 ng/mL. Complement C3 was 0.74 g/L, complement C4 was 0.11 g/L, and urinalysis showed proteinuria (2+). Chest computed tomography demonstrated scattered inflammatory changes in both lungs, with partial consolidation in the lower lobes.

### 2.7. Diagnostic reasoning

At that stage, hypotension, fever, thrombocytopenia, elevated PCT, and inflammatory abnormalities on imaging made initial treatment for septic shock reasonable. As the course evolved, however, the timing of antivenom exposure, the generalized urticarial and purpuric rash, decreased complement levels, proteinuria, and later recovery pattern increasingly supported a severe delayed serum sickness-like reaction as an important contributor. Because systematic pathogen testing was not completed during the acute phase, infection could not be fully excluded. The clinical diagnosis was therefore a delayed serum sickness-like reaction after antivenom therapy that mimicked septic shock, with suspected infection treated concurrently.

### 2.8. Therapeutic intervention during the second hospitalization

The patient was initially treated as having suspected sepsis and septic shock, with empirical anti-infective therapy and hemodynamic support. Vasopressor support was started with norepinephrine at 0.03 μg/kg/min and later changed to metaraminol at 0.83 μg/kg/min for a total of 3 days. Antimicrobial treatment consisted of piperacillin-tazobactam 4.5 g 3 times daily for 7 days and levofloxacin 0.75 g once daily for 6 days. For the suspected severe serum sickness-like reaction, hydrocortisone 100 mg/d was administered for 3 days and ebastine 10 mg once daily was given for 7 days. Supportive treatment included fluid replacement, gastric protection, electrolyte management, measures to improve vascular permeability, diuretics when necessary, and albumin supplementation. Endocrine and rheumatologic/immunologic evaluations were also performed during hospitalization.

### 2.9. Follow-up and outcomes

After 7 days of treatment, blood pressure stabilized, dizziness and fatigue improved, and rash and pruritus largely resolved. Platelet count and inflammatory markers gradually improved. She was discharged on September 9, 2025. At outpatient follow-up on September 24, 2025, the white blood cell count, neutrophil percentage, platelet count, hs-CRP, serum amyloid A, albumin level, and thyroid function had improved substantially compared with previous values. The overall clinical course is summarized in the separately uploaded Table 1, and trends in inflammatory and hematologic parameters are shown in Figure 1.

**Table 1:** Clinical timeline and major management steps.

Date	Time from bite/antivenom	Main events
August 24, 2025	Approximately 1 h after bite	First admission because of pain and swelling of the left hand.
August 24, 2025	Within 1 h after admission	Two vials of *Deinagkistrodon acutus* antivenom administered intravenously; no immediate hypersensitivity reaction.
August 28, 2025	4 d after first admission	Discharged after improvement in local swelling and pain.
August 31, 2025	Approximately 7 d after antivenom	Generalized urticarial rash, pruritus, vomiting, poor appetite, and fatigue appeared.
September 1, 2025	Approximately 8 d after antivenom	Returned to the emergency department; rash and pruritus worsened; fever reached 39.3°C.
September 1–2, 2025	Approximately 8–9 d after antivenom	Dizziness, hypotension, and bradycardia occurred; vasopressor support was required.
September 2, 2025	Approximately 9 d after antivenom	Second admission with suspected serum sickness and shock of undetermined cause.
September 2–9, 2025	Second hospitalization	Empirical anti-infective treatment, hemodynamic support, hydrocortisone, ebastine, albumin supplementation, and supportive care.
September 9, 2025	Discharge	Blood pressure stabilized; rash and pruritus largely resolved; inflammatory markers and platelet count improved.
September 24, 2025	Outpatient follow-up	Blood counts, inflammatory markers, albumin level, and thyroid function had improved substantially.

Antivenom timing refers to the interval from antivenom administration.

**Figure 1.**
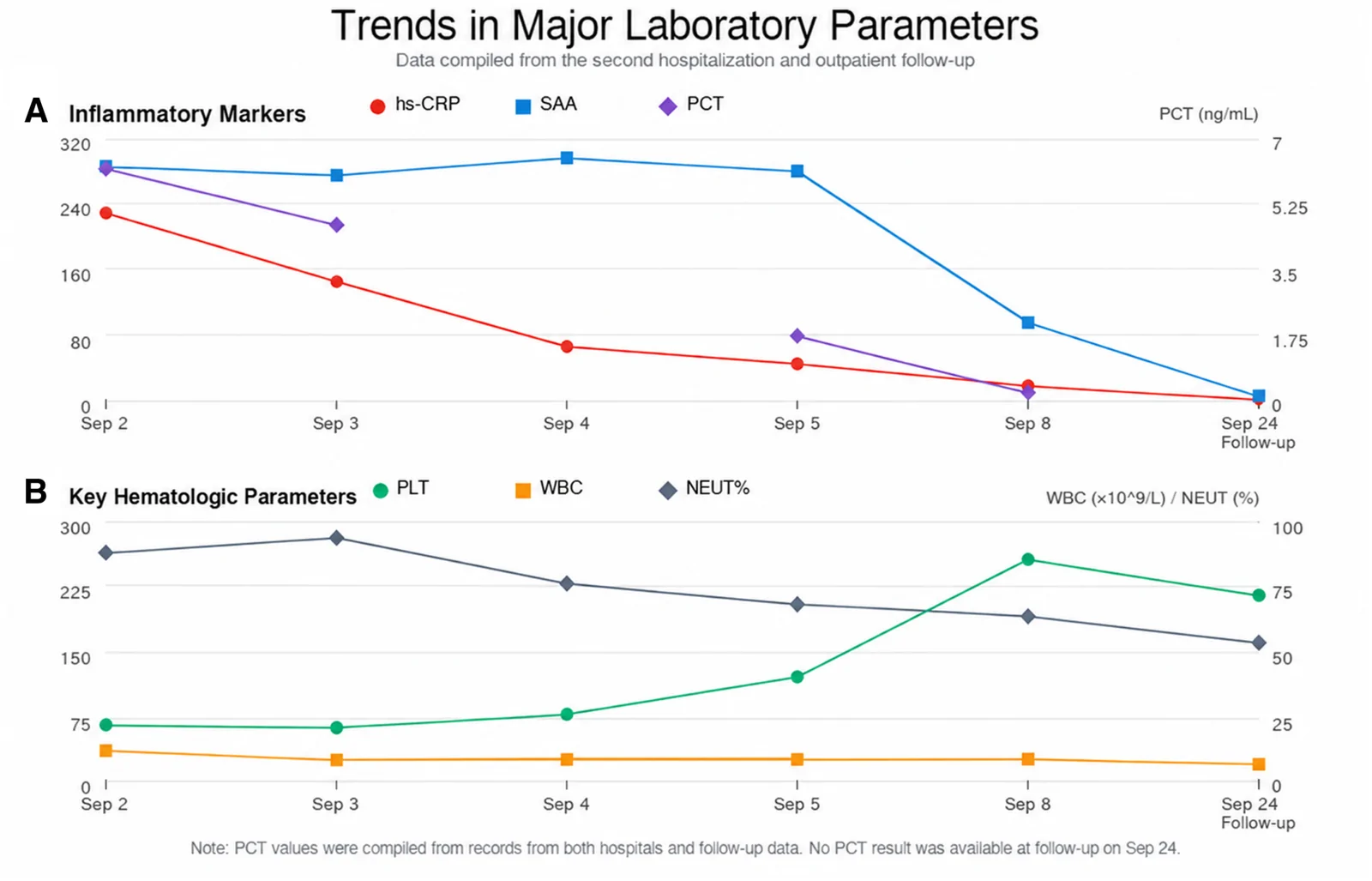
Trends in inflammatory (A) and hematologic parameters (B) during the second hospitalization and follow-up. The figure highlights the decline in inflammatory markers and recovery of hematologic parameters after empirical anti-infective therapy, hemodynamic support, glucocorticoid treatment, antihistamine therapy, and supportive care. hs-CRP = high-sensitivity C-reactive protein, NEUT% = neutrophil percentage, PCT = procalcitonin, PLT = platelet count, SAA = serum amyloid A, WBC = white blood cell count.

## 3. Discussion

This case is clinically important because it demonstrates how a delayed antivenom-related serum sickness-like reaction can present as a shock syndrome with laboratory features that strongly suggest infection. The initial emergency management was appropriate because the patient had fever, hypotension requiring vasopressor support, thrombocytopenia, markedly elevated hs-CRP, elevated PCT, and inflammatory abnormalities on chest computed tomography.^[8–10]^ In such a setting, delayed treatment of possible sepsis could be harmful. The main lesson is not that infection should be dismissed, but that clinicians should continue to reassess the diagnosis when the clinical pattern contains immune-mediated clues after antivenom exposure.

The timing and immune phenotype were central to the diagnostic reassessment. Serum sickness after antivenom commonly occurs several days after exposure and may manifest with rash, fever, pruritus, arthralgia, lymphadenopathy, proteinuria, and systemic symptoms. In this patient, generalized pruritic urticarial and purpuric maculopapular eruptions appeared approximately 1 week after antivenom administration. Complement C3 and C4 were decreased, and urinalysis showed proteinuria. These findings supported immune-complex-mediated inflammation or a serum sickness-like reaction. The subsequent improvement after antihistamine therapy, glucocorticoids, and supportive care further supported this interpretation, although concurrent infection could not be fully excluded because microbiological testing was incomplete.

PCT was the major confounding factor in this case. PCT is commonly used to support the assessment of bacterial infection and sepsis, but it is not a stand-alone diagnostic test. Severe systemic inflammation, shock, tissue injury, and noninfectious inflammatory conditions may also be associated with increased PCT. Therefore, elevated PCT should be interpreted together with exposure history, physical findings, microbiological evidence, imaging findings, complement levels, urinalysis, and the subsequent clinical course. For patients recently treated with antivenom, the presence of a typical delayed rash, complement consumption, and urinary abnormalities should prompt consideration of a serum sickness-like reaction even when PCT is increased.

This case also has practical implications for discharge education and follow-up after antivenom therapy. Patients are often discharged once local swelling, pain, or coagulation abnormalities improve. However, delayed immune reactions may develop after discharge, usually within the first 1 to 2 weeks. Patients and primary care clinicians should be advised to seek medical reassessment promptly if rash, fever, pruritus, dizziness, hypotension, edema, marked fatigue, or urinary abnormalities occur after antivenom treatment. Directly asking about recent antivenom exposure may prevent misclassification of these cases as infection alone.

The report has several limitations. First, the biting snake was not captured, and the suspected *P mucrosquamatus* envenoming was based on epidemiological background, local manifestations, and laboratory findings rather than taxonomic confirmation. Second, systematic pathogen testing was not completed during the acute phase, so infection could not be excluded on an evidentiary basis. Third, this is a single case report, and some details are limited by the completeness of the original medical record. These limitations are why we describe the event as a serum sickness-like reaction that mimicked septic shock, rather than as a proven noninfectious shock syndrome.

## 4. Conclusion

Delayed serum sickness-like reactions after antivenom therapy may present with fever, rash, circulatory instability, thrombocytopenia, elevated inflammatory markers, and elevated PCT, thereby mimicking septic shock. In patients who develop rash and hypotension within days after antivenom treatment, clinicians should treat possible infection promptly while actively reassessing the timing of antivenom exposure, skin findings, complement levels, urinary abnormalities, microbiological evidence, and response to anti-allergic or glucocorticoid therapy.

## Acknowledgments

The authors thank the clinical team involved in the diagnosis, treatment, and follow-up of this patient. OpenAI ChatGPT/Codex was used to assist with English language editing, manuscript formatting, and adaptation of the case report to the target journal style based on author-provided clinical information. The authors reviewed, revised, and verified the entire manuscript, including all clinical content, interpretations, references, and declarations, and take full responsibility for the accuracy and integrity of the submitted work.

## Author contributions

**Data curation:** Xiaobin Wang.

**Formal analysis:** Xiaobin Wang, Wenkai Bin.

**Resources:** Xiaobin Wang.

**Conceptualization:** Wenkai Bin.

**Investigation:** Wenkai Bin.

**Methodology:** Wenkai Bin.

**Project administration:** Wenkai Bin.

**Supervision:** Wenkai Bin.

**Validation:** Wenkai Bin.

**Writing – original draft:** Xiaobin Wang.

**Writing – review & editing:** Xiaobin Wang.
